# Supplementary material for: Equilibrium shift, poisoning prevention, and selectivity enhancement in catalysis via dehydration of polymeric membranes
Source: Nat Commun. 2023 Mar 25;14:1673. doi: 10.1038/s41467-023-37298-y (PMC10039873; doi:10.1038/s41467-023-37298-y)
Supplement: Supplementary file 1 — Supplementary Information [file 41467_2023_37298_MOESM1_ESM.pdf]

Supplementary Information

Equilibrium shift, poisoning prevention, and selectivity enhancement in catalysis via dehydration of polymeric membranes

This PDF file includes:

Supplementary Methods
Supplementary Figure 1–16
Supplementary Table 1–2
Supplementary References

Supplementary Methods

Catalysts

A commercially available $CuO/ZnO/Al_2O_3$ catalyst (ShiftMax210, Clariant) was used for the reverse water–gas shift (RWGS) reaction. An alumina-supported palladium catalyst (1 wt%. Pd/γ-Al_2O_3) for methane oxidation was prepared using the impregnation method. The gamma alumina support (Puralox Scca-5/170, Sasol) was impregnated with an aqueous palladium(II) nitrate dihydrate (40% Pd) (Sigma-Aldrich) solution, and the suspension was stirred for 2 h. Excess water was removed using a rotary evaporator at 50 °C, and the catalyst was dried at 100 °C for 12 h and calcined at 500 °C for 5 h. The catalyst for the Fischer–Tropsch olefin (FTO) synthesis, $K_4Fe_{100}Cu_6Al_{16}$, was prepared by the coprecipitation of Fe–Cu–Al precursors, followed by a potassium wetness impregnation method [1].

Membrane

Monomers 4,4′-hexafluoroisopropylidene diphthalic anhydride (6FDA, >99%, Daikin Industries Ltd., Japan), 2,2-bis(3-amino-4-hydroxyphenyl)hexafluoropropane (bisAPAF, > 99%, Merck, Germany), and 3,3′-dihydroxy-4,4′-diamino-biphenyl (HAB, >99%, Changzhou Sunlight Pharmaceutical Co., Ltd., China) were used for the synthesis of the hydroxyl polyimide (HPI) precursor. Reagents *N*-methyl-2-pyrrolidone (NMP, 99.5%), tetrahydrofuran (THF, 99.5%), and *o*-xylene (98.0%) were purchased from Samchun Pure Chemicals (Korea). All reagents and monomers were used without further purification.

Synthesis of hydroxyl polyimide (HPI) precursor

As shown in **Supplementary Fig. 2a**, the HPI precursor was synthesized by the azeotropic imidization method using 6FDA (250 mmol, 111.060 g), bisAPAF (125 mmol, 45.783 g), and HAB, (125 mmol, 27.030 g) (molar ratio of the three components was 1:0.5:0.5). Two diamines, bisAPAF and HAB, were dissolved in NMP (1010 mL) and mechanically stirred for 2 h under an N_2-purged atmosphere. Subsequently, 6FDA was carefully added to the diamine mixture at 0 °C and stirred for 12 h to synthesize the poly(amic acid) (PAAc) intermediate. After the reaction, a viscous brown-colored PAAc solution was obtained. For the imidization step, *o*-xylene (1010 mL) as an azeotropic agent was added to the PAAc solution and refluxed at 180 °C for 18 h with vigorous stirring in a heating mantle. The water generated during the conversion from the amic acid groups to imide rings was evaporated as an azeotrope with *o*-xylene and trapped in a Dean–Stark trap connected to a cooled condenser. The resulting dark brown HPI solution was cooled to ambient temperature and ground with DI water in an electric blender for precipitation. The HPI precursor was dried in a vacuum oven at 80 °C after several washing steps. Finally, 131.05 g of the HPI precursor powder was obtained, corresponding to 83% yield.

Fabrication of HPI precursor hollow fibers

The detailed doping composition for hollow fiber spinning is given in **Supplementary Table 1**. HPI (50 g) was completely dissolved in a mixture of 134.78 g NMP and 32.60 g THF in a dope reservoir by mechanical stirring for 12 h. Then, the reservoir was sealed and kept inside an oven at 60 °C for 2 h to degas microbubbles. If necessary, further degassing was performed using a

vacuum pump. Spinning was conducted by a dry-jet/wet-quench method using home-built spinning setup (**Supplementary Fig. 3**) under the conditions listed in **Supplementary Table 1**. More details on the spinning procedure can be found in our previous study [2]. The spun HPI fibers were rinsed in a washing bath filled with tap water for 24 h, and then dried in air for 12 h. The average diameter and wall thickness of the HPI fibers were 508.2 and 54.4 μm, respectively (**Supplementary Fig. 6a**), as evaluated by SEM.

Single gas and water vapor permeation test at high temperature in TR-PBO fiber module

The gas separation performance of the TR-PBO fiber module was evaluated in a dead-end (feed-out closed) system using single gases, viz. H_2, CO_2, CO, and CH_4, in the temperature range of 200–400 °C. The test setup is illustrated in **Supplementary Fig. 10**; the membrane temperature was controlled using a customized furnace. Each gas was pressurized to 0.2 MPa at the shell side of the TR-PBO fibers in the module, and the permeate gas flow rate out of the bore side of the fibers was measured using a soap-bubble flowmeter.
The single gas permeance was calculated using **(1)**:

$$\text{Gas permeance } (P_{\text{gas}}) = \frac{Q}{A \cdot \Delta p} \qquad \text{equation (1).}$$

Here, P_{gas} is the gas permeance, Q is the permeate gas flow rate (cm^3/s), A is the effective membrane area (cm^2), and p is the applied feed pressure (cm Hg).
The water vapor permeance was determined from the difference in relative humidity (RH) between the feed and permeate sides. A N_2 stream as a carrier gas was provided at the feed side of the TR-PBO module, and the feed pressure was controlled at 2 bar by a back-pressure regulator at the retentate side. Water vapor was generated using a customized vapor generator at 350 °C and supplied to the N_2 stream at the feed side. The permeate flow rate of water vapor (Q_{H2O}) was determined from the difference in the N_2 flow rate with and without water vapor on the permeate side, which was measured using a soap-bubble flowmeter. The permeate N_2 flow rate without water vapor was determined by measuring the N_2 stream passing through a water trap in an electric chiller. The RH at the given temperature was measured using an electric hygrometer (635-2, Testo, Germany) at the feed and permeate sides.
To calculate the water vapor permeance, the partial pressure of water vapor (p_{H2O}) at the feed and permeate sides was obtained using the following relationship:

$$\frac{p_{H_2O}}{p_{sat}} = \frac{\text{RH}(\%)}{100} \qquad \text{equation (2).}$$

Here, p_{sat} is the saturated vapor pressure of water at a given measurement temperature.
The water vapor permeance was calculated using **(3)**:

$$\text{Water vapor permeance } (P_{H_2O}) = \frac{Q_{H_2O}}{A \cdot \Delta p_{H_2O}} \qquad \text{equation (3).}$$

Here, $\Delta p_{,H2O}$ is the difference between the partial pressure of the water vapor in the feed and permeate sides.
Water vapor/gas permselectivity was simply calculated using **(4)**:

$$H_2O/\text{Gas permselectivity} = \frac{p_{H_2O}}{P_{gas}} \qquad \text{equation (4).}$$

The determined permeance and permselectivity of the single gases and water vapor are listed in **Supplementary Table 2**.

Reaction 1: Reverse Water–Gas Shift

As shown in **Supplementary Fig. 12a**, the RWGS reaction using the neat catalyst was conducted in the temperature range of 250–425 °C, and the product gases at the feed-out side were characterized by an electric mass flowmeter (MFM) and gas chromatography (GC). For each data point at a different temperature, the temperature was maintained for 8 h before data acquisition at a steady state. N_2 gas was used as an internal standard gas for GC analysis and was injected at a flow rate of 12.0 sccm just behind the feed-out side.
Because of the negligible side reactions, the CO yield at the steady state could be calculated directly based on the CO_2 conversion:

$$CO_2 \text{ conversion } (\%) = \frac{\text{Amount of } CO_2 \text{ converted}}{\text{Amount of } CO_2 \text{ passed through the catalyst bed}} \times 100 \qquad \text{equation (5).}$$

The equation can be expanded as follows:

$$CO_2 \text{ conversion } (\%) = \frac{F_{C,in} \times C_{C,in} - F_{C,out} \times C_{C,out}}{F_{C,in} \times C_{C,in}} \times 100 \qquad \text{equation (6).}$$

Here, $F_{C.in}$ and $F_{C,out}$ are the flow rates of the feed-in and feed-out sides, respectively. $C_{C,in}$ and $C_{C,out}$ refer to the CO_2 compositions at the feed-in and feed-out sides, respectively.
For the RWGS reaction in the membrane module, the conversion was calculated using an approach different from the without-membrane reaction equation.

The CO_2 conversion can be calculated as follows:

$$CO_2 \text{ conversion } (\%) = \frac{F_{R.in} \times C_{R.in} + F_{S.in} \times C_{S.in} - (F_{R.out} \times C_{R.out} + F_{S.out} \times C_{S.out})}{F_{R.in} \times C_{R.in} + (F_{S.in} \times C_{S.in} - F_{S.out} \times C_{S.out})} \times 100 \qquad \text{equation (6).}$$

Here, the denominator contains the initial amount of CO_2 fed into the feed-in side and the CO_2 quantity passed across the membrane from the sweep side to the reaction side during the reaction. $F_{R,in}$, $F_{R,out}$, $F_{S,in}$, and $F_{S,out}$ refer to the flow rates on the feed-in, feed-out, sweep-in, and sweep-out sides, respectively. Characterized by GC analysis, $C_{R,in}$, $C_{R,out}$, $C_{S,in}$, and $C_{S,out}$ refer to the CO_2 concentrations at the feed-in, feed-out, sweep-in, and sweep-out sides, respectively.

The carbon balance of the RWGS shift was calculated as follows:

$$\text{C balance}(\%) = \frac{\text{Amounts of } CO_2 \text{ and CO detected out of the reactor}}{\text{Amount of } CO_2 \text{ fed into the reactor}} \times 100 \qquad \text{equation (7).}$$

The amount of water produced per unit time at a relevant temperature was estimated using the RH values measured using digital hygrometers at the feed-out and sweep-out sides. For calculation, the RH values were converted to absolute humidity (H_a) using a psychrometric chart. Then, the amount of water per unit time was calculated using the following relation:

$$\text{Amount of water produced per unit of time (mg/min)} = H_a \cdot \rho_{gas} \cdot Q_{gas} \qquad \text{equation (8)}$$

Here, H_a is the mass of water vapor per unit mass of dry gas, ρ_{gas} is the density of dry gas, and Q_{gas} is the volume flow rate.

Reaction 2: Methane Combustion

The CH_4 conversion is defined below

$$CH_4 \text{ conversion } (\%) = \frac{\text{Amount of } CH_4 \text{ converted}}{\text{Amount of } CH_4 \text{ passed through the catalyst bed}} \times 100 \qquad \text{equation (9).}$$

The equation can be expanded as

$$CH_4 \text{ conversion } (\%) = \frac{F_{R.in} \times C_{R.in} - F_{R.out} \times C_{R.out}}{F_{R.in} \times C_{R.in}} \times 100 \qquad \text{equation (10).}$$

Here, $F_{C.in}$ and $F_{C,out}$ refer to the flow rates of the feed-in and feed-out sides, respectively, while $C_{C,in}$ and $C_{C,out}$ refer to the CH_4 compositions at the feed-in and feed-out sides, respectively.

The carbon balance of the CH_4 oxidation was calculated as follows:

$$\text{C balance}(\%) = \frac{\text{Amounts of } CH_4 \text{ and } CO_2 \text{ detected out of the reactor}}{\text{Amount of } CH_4 \text{ fed into the reactor}} \times 100 \qquad \text{equation (11).}$$

The CH_4 conversion was calculated using **(9)** because the amount of gas exchanged through the membranes was negligible. The amount of water produced was measured using hygrometers installed on both sides and was calculated using **(8)**.

We note that since the concentration of methane used in the methane oxidation reaction is low (0.4%), there was a slight experimental error in measuring the absolute amount of unreacted methane and carbon dioxide generated after the reaction. A multiposition valve was installed at the rear end of the reactor when using a membrane for methane oxidation to alternately measure the two outlet gases of the reaction part and the sweep part, which resulted in a slight measurement error during operation.

Reaction 3: Fischer–Tropsch Olefin Synthesis

The CO conversion is defined by **(12)**:

$$\text{CO conversion}(\%) = \frac{\text{Amount of CO converted}}{\text{Amout ofCO passed through the catalyst bed}} \times 100 \qquad \text{equation (12).}$$

The equation can be expanded as

$$\text{CO conversion }(\%) = \frac{F_{R.in}\times C_{R.in}+F_{S.in}\times C_{S.in}-(F_{R.out}\times C_{R.out}+F_{S.out}\times C_{S.out})}{F_{R.in}\times C_{R.in}+(F_{S.in}\times C_{S.in}-F_{S.out}\times C_{S.out})} \times 100 \qquad \text{equation (13).}$$

Here, $F_{R,in}$, $F_{R,out}$, $F_{S,in}$, and $F_{S,out}$ are the flow rates at the feed-in, feed-out, sweep-in, and sweep-out sides, respectively. Characterized by GC analysis, $C_{R,in}$, $C_{R,out}$, $C_{S,in}$, and $C_{S,out}$ refer to the CO concentrations at the feed-in, feed-out, sweep-in, and sweep-out sides, respectively.

The selectivity of the Fischer–Tropsch reaction was calculated as

$$\text{CO}_2\text{ selectivity}(\%) = \frac{\text{Amount of CO}_2\text{ produced}}{\text{Amount of CO converted}} \times 100 \qquad \text{equation (14),}$$

$$\text{C}_{1-4}\text{ selectivity }(\%) = \frac{\text{Amount of C}_{1-4}\text{ hydrocarbons produced}}{\text{Amount of converted CO}} \times 100 \qquad \text{equation (15),}$$

$$\text{C}_{5+}\text{ hydrocarbons selectivity }(\%) = 100 - \text{C balance} \qquad \text{equation (16).}$$

The carbon balance of the Fischer–Tropsch olefin synthesis was calculated using

$$\text{C balance}(\%) = \frac{\text{Amounts of CO,CO}_2\text{,C}_{1-4}\text{detected out of the reactor}}{\text{Amount of CO fed into the reactor}} \times 100 \qquad \text{equation (17).}$$

Thermogravimetric analysis

The thermal rearrangement temperature of the HPI precursor hollow fibers and the thermal degradation (T_d) temperature of the TR-PBO hollow fibers were determined from the weight loss curves obtained by thermogravimetric analysis (TGA; TGA-N1000, Scinco M&T, Korea). For the HPI and TR-PBO fiber samples, the heating protocol included the following steps: heating up to 300 °C at 5 °C/min (Step 1), dwelling for 1 h to remove residual solvents (Step 2), heating up to 800 °C (Step 3), and cooling down to ambient temperature (Step 4). The weight loss curves of TR-PBO fibers were obtained under two different purge gases, N_2 and O_2 (99.99%, Chungang Industry Gas, Korea) streams, at a flowrate of 40 ccm. As seen in **Supplementary Fig. 4a**, two-step weight loss was observed at 300–430 °C and beyond 430 °C. In general, the HPI precursor

undergoes sequential thermal weight loss due to the thermal rearrangement and thermal degradation [3]. Thus, the temperature range of the thermal rearrangement of the HPI precursor was 320–450 °C. The heating protocol for the thermal rearrangement involved heating to 425 °C for 30 min, after which the thermal rearrangement was above 80% based on the weight loss curve in **Supplementary Fig. 4b**. The residual weight loss reached completion during the cooling step, achieving approximately 100% conversion. The weight loss region in the TGA isotherm at 400 °C (**Supplementary Fig. 9)** confirmed <0.15% weight reduction over 4 h under a N_2 atmosphere.

Differential scanning calorimetry

The glass transition temperature (T_g) of the TR-PBO hollow fibers was observed using a differential scanning calorimetry (DSC) instrument (Thermo plus EVO II DSC 8230, METTLER TOLEDO, Switzerland). Under a N_2 atmosphere, the heating cycle was carried out by heating up to 400 °C at a rate of 10 °C/min. As seen in **Supplementary Fig. 8**, the T_g of the TR-PBO fibers could not confirmed, unlike the HPI precursor fibers reported previously **[2]**. The relevant supplementary data can be found in **Supplementary Fig. 8**.

Field-emission scanning electron microscopy

The cross-sectional morphologies of the HPI precursor and TR-PBO fibers were observed using a field-emission scanning electron microscope (FE-SEM, TESCAN MIRA3 LMU, TESCAN Ltd., Czech Republic).

X-ray diffraction

The changes in the interchain distance and *d*-spacing in the TR-PBO dense films at high temperatures were estimated from the 2θ values in the wide-angle X-ray diffraction (XRD; Rigaku Ultima IV, Japan) patterns. Under a N_2 atmosphere, XRD patterns were obtained at a scanning rate of 2°/min at 250, 300, 350, and 400 °C. The *d*-spacing values were then calculated from the 2θ value at the maximum intensity using Bragg's equation [4]

Attenuated total reflectance infrared spectroscopy

The synthesized HPI precursor and TR-PBO were structurally identified by attenuated total reflectance infrared (ATR-IR) spectroscopy (ALPHA-P FT-IR spectrometer, Bruker, USA). The relevant supplementary data can be found in **Supplementary Fig. 7** [5].

X-ray photoelectron spectroscopy

The catalysts before and after the target reactions were characterized by XPS (AXIS SUPRA, KRATOS, UK) using a monochromated Al Kα X-ray source (hν = 15 keV). Valence band photoemission spectra were obtained at a pass energy of 20 eV.

Gas chromatography

The effluent gas from the reactor was analyzed on a gas chromatograph (YL6500 GC, YL Instruments, Korea) equipped with a TCD and FID. The gas products, H_2, CO, CH_4, and CO_2, and the internal N_2 gas were analyzed using GC-TCD with Carboxen 1000, and the hydrocarbon products (C1–C4) were analyzed using GC-FID with a GS-GASPRO column. The column temperature was maintained at 200 °C and the TCD and FID temperatures were maintained at 150 and 250 °C, respectively. To initialize the GC instrument, the system temperature was programmed to stand at 50 °C for 10 min and then ramped up to 190 °C at 20 °C/min. Ar (100.00%, Chungang Industry Gas, Korea) was used as the carrier gas at 30 cc/min for TCD and 3 cc/min for FID, respectively.

Supplementary Figures

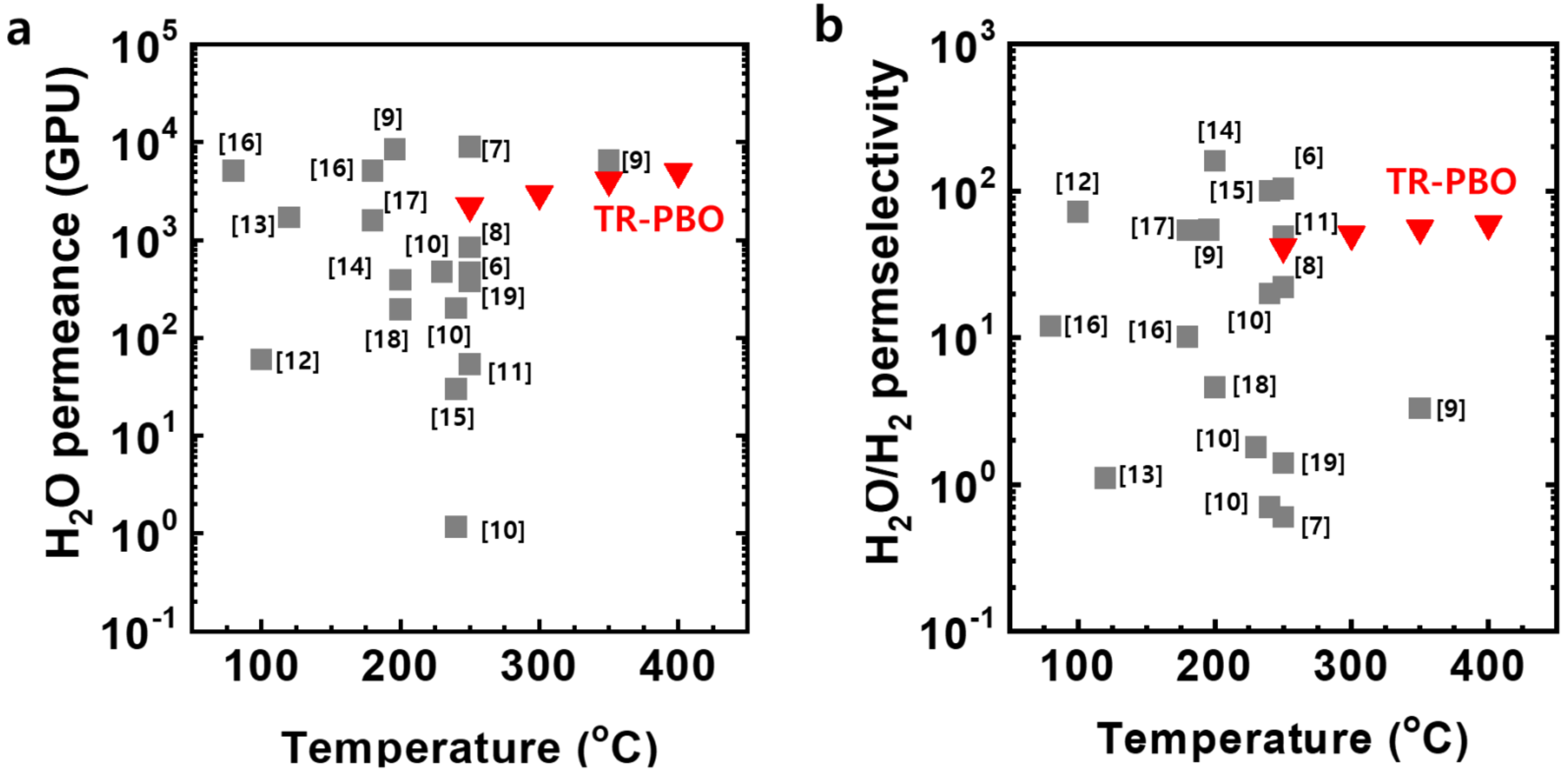

Supplementary Fig. 1. a Water vapor permeance and **b** H_2/H_2O selectivity of inorganic membranes at 250–400 °C (6-19). The numbers in the panels indicate supplementary reference numbers: [6, NaA zeolite membrane], [7, MFI zeolite membrane], [8, NAZSM-5 membrane], [9, ZSM-5 membrane], [10, Zeolite membrane], [11, mordenite membrane],[12, Zeolite 4A membrane], [13, NaA zeolite membrane], [14, A-type zeolite membrane], [15, Zeolite A membrane], [16, ZSM-5 membrane], [17, FAU-type zeolite membrane], [18, SOD and LTA membrane], and [19, SOD membrane].

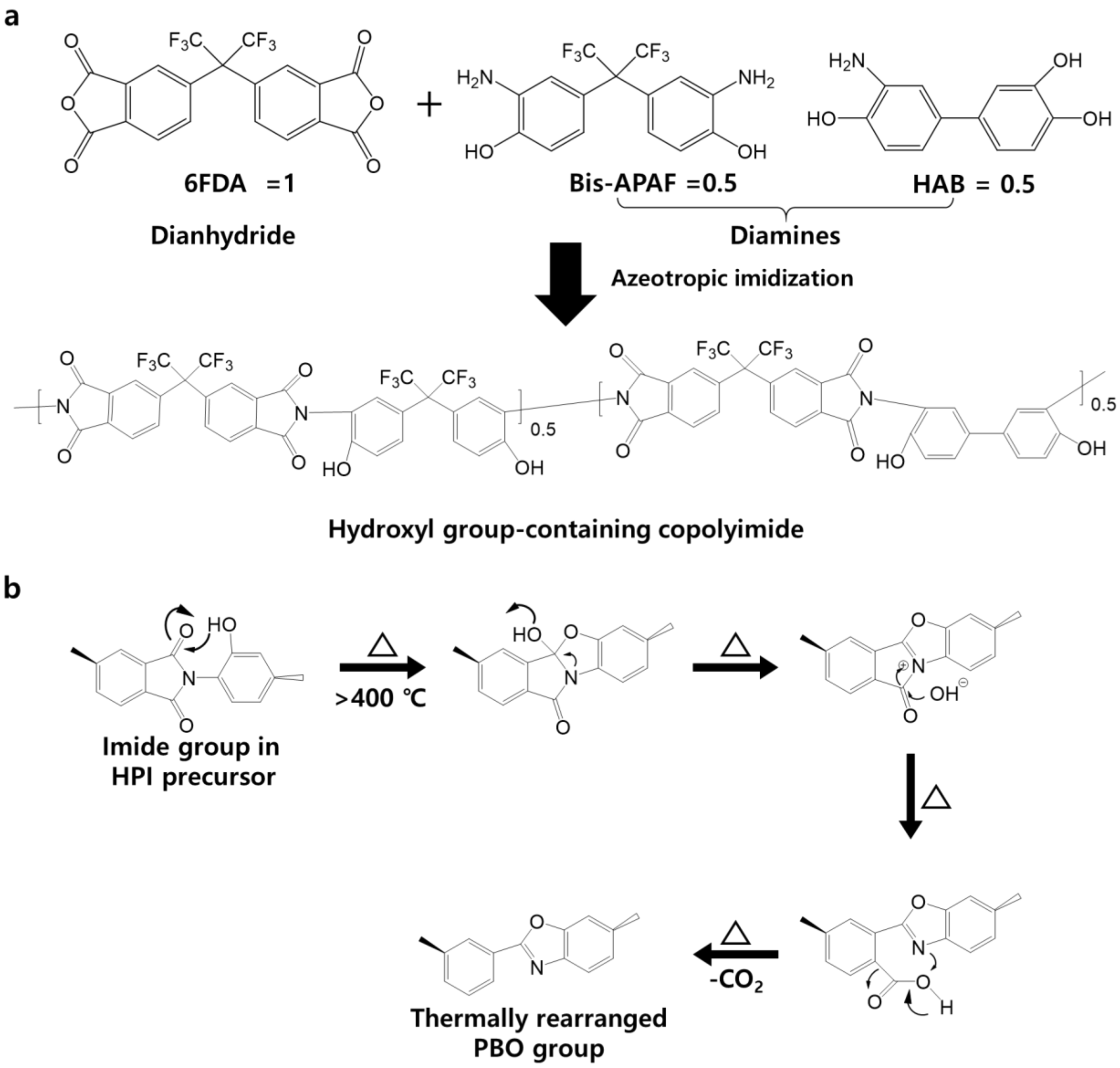

Supplementary Fig. 2. a Synthesis of HPI precursor and **b** its thermal rearrangement to TR-PBO.

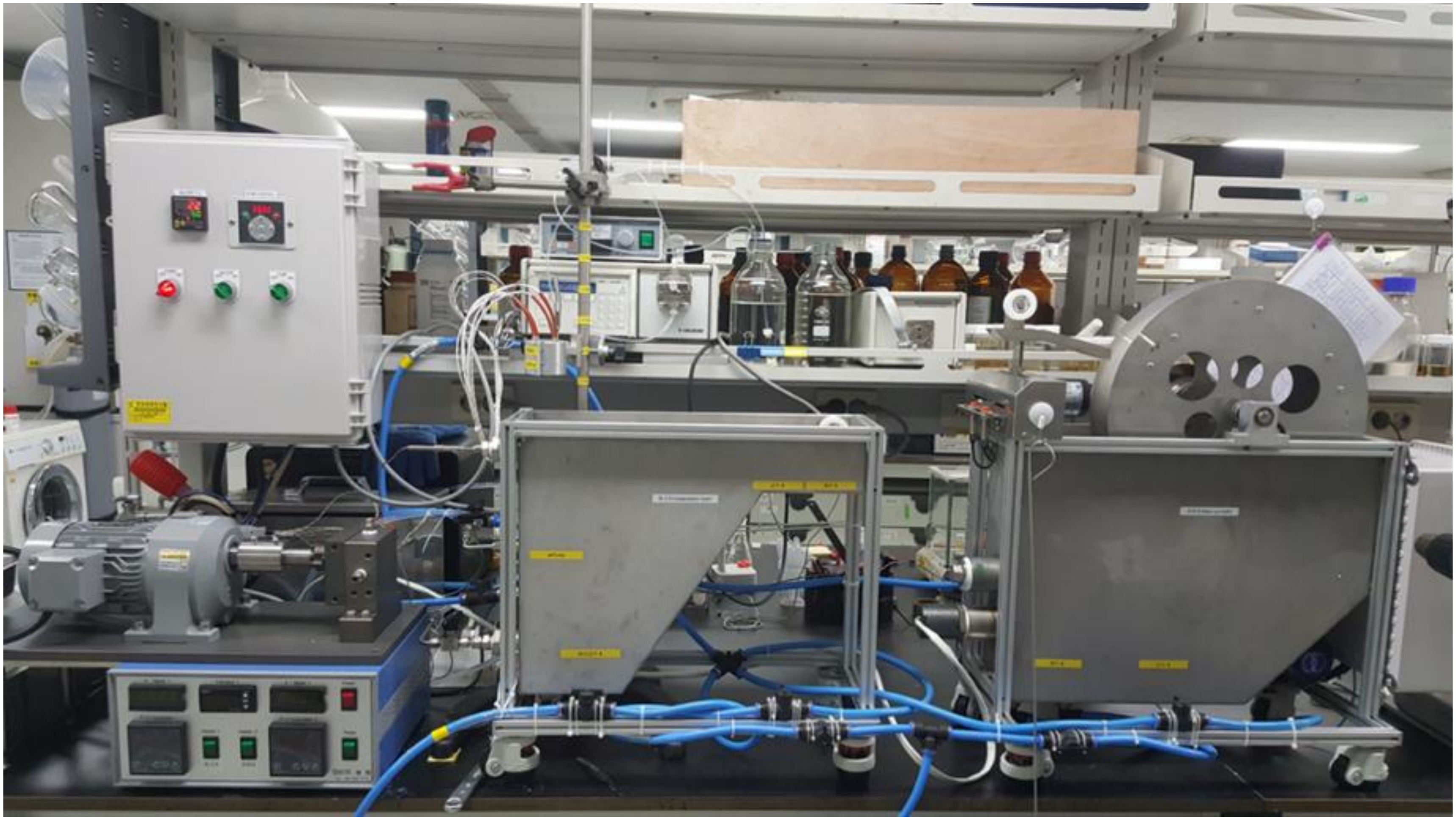

Supplementary Fig. 3. Home-built hollow fiber spinning setup

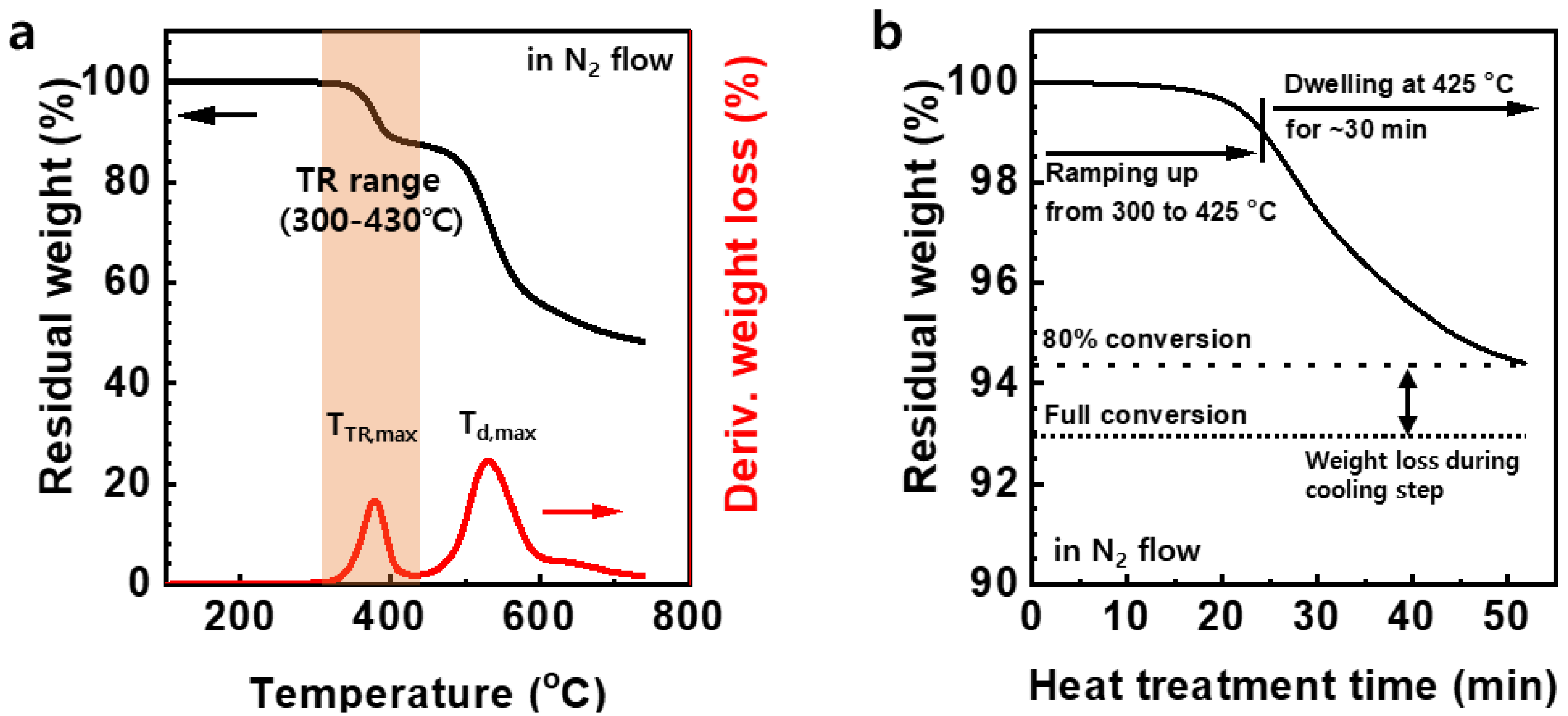

Supplementary Fig. 4. Thermogravimetric analysis of HPI precursor: **a** TGA graph of HPI precursor in the range of 100–800 °C, **b** HPI heating protocol to form TR-PBO

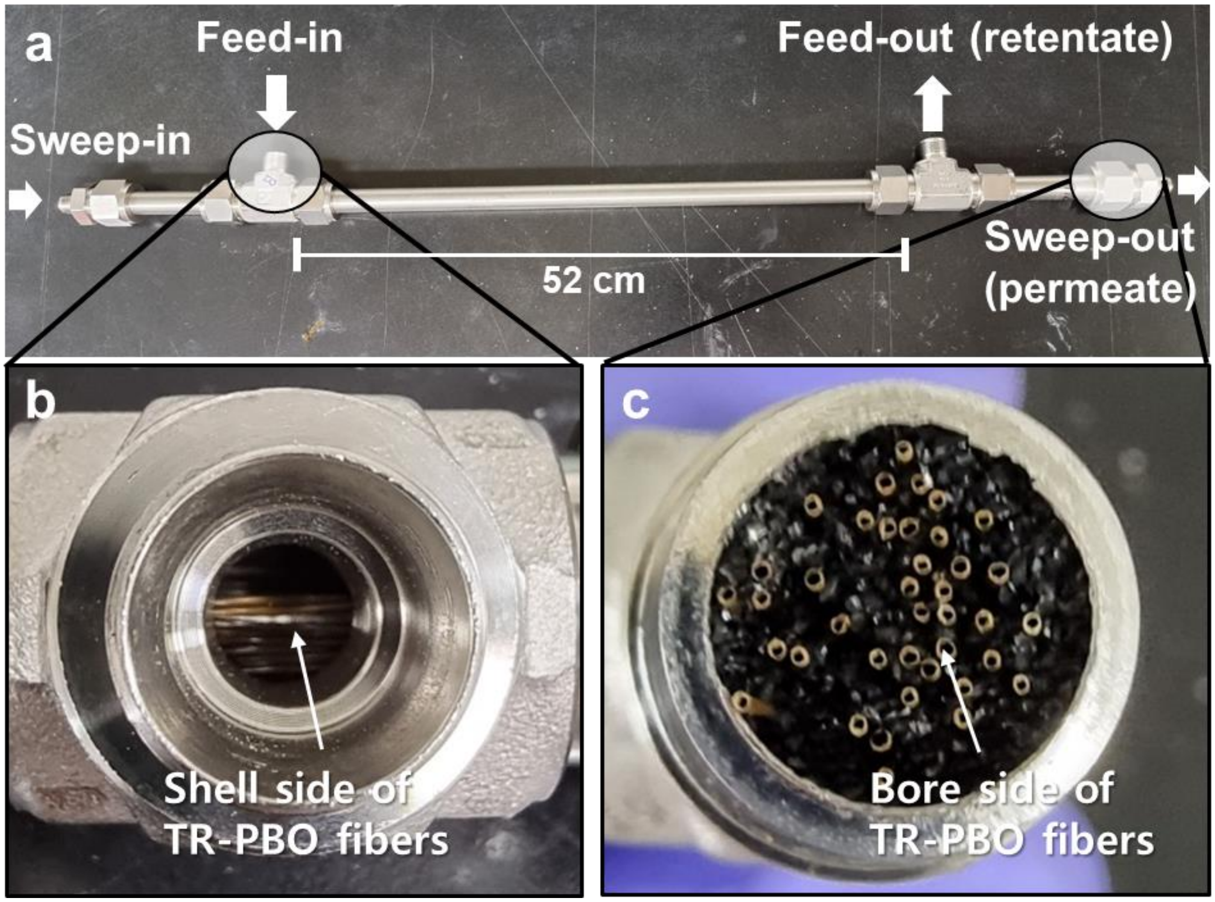

Supplementary Fig. 5. Photograph of the TR-PBO HF module in this study: **a** overall shape, **b** shell side (feed side), and **c** bore side (permeate side)

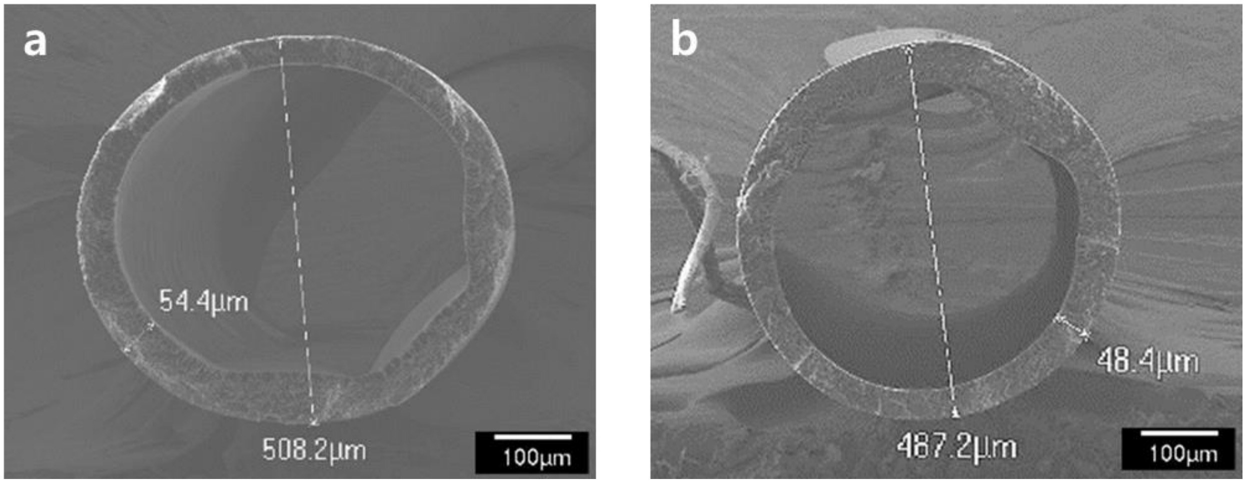

Supplementary Fig. 6. SEM images of **a** HPI and **b** TR-PBO hollow fiber membrane used in this study

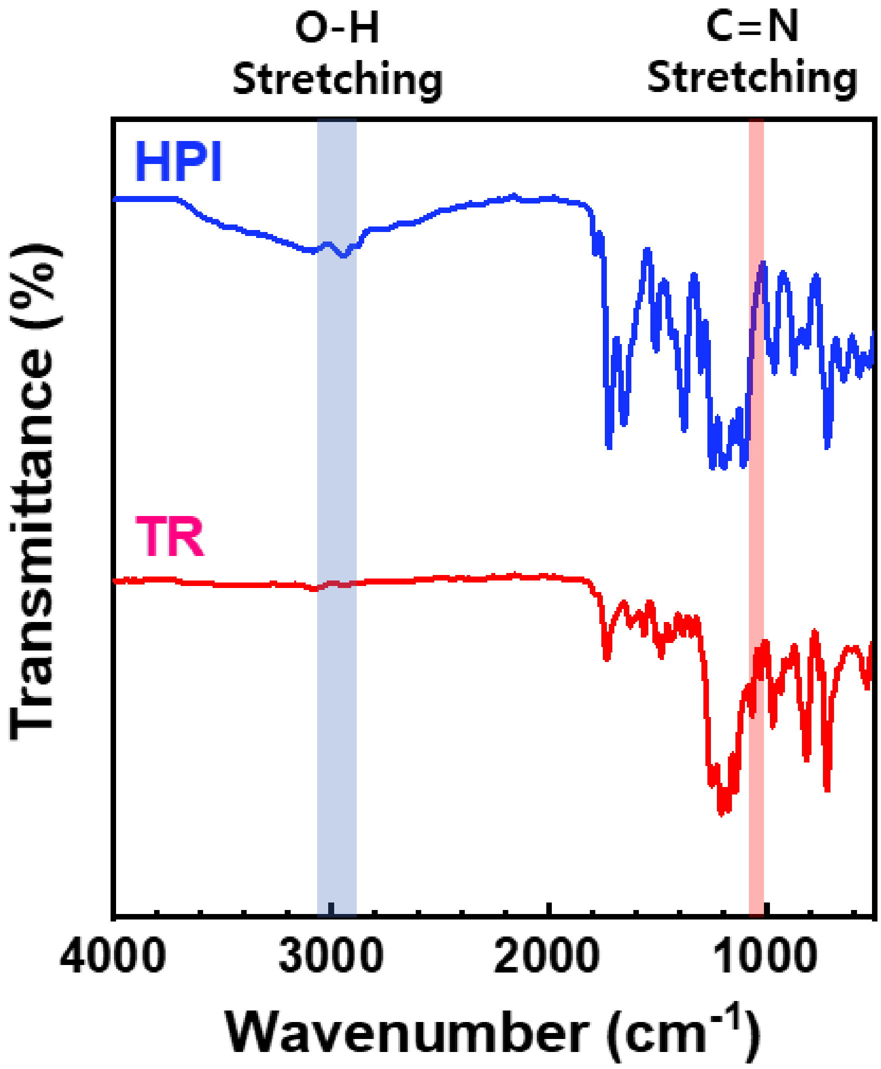

Supplementary Fig. 7. FTIR spectra of the HPI precursor and TR-PBO film

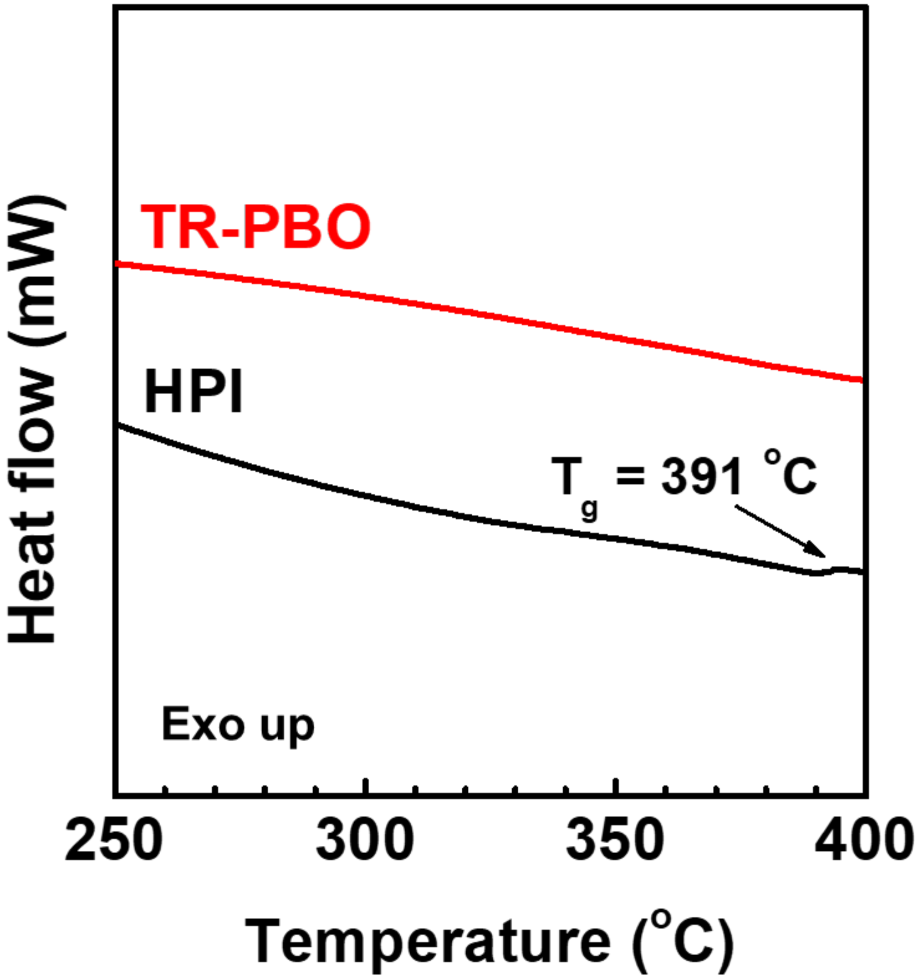

Supplementary Fig. 8. DSC curve of TR-PBO and HPI precursor

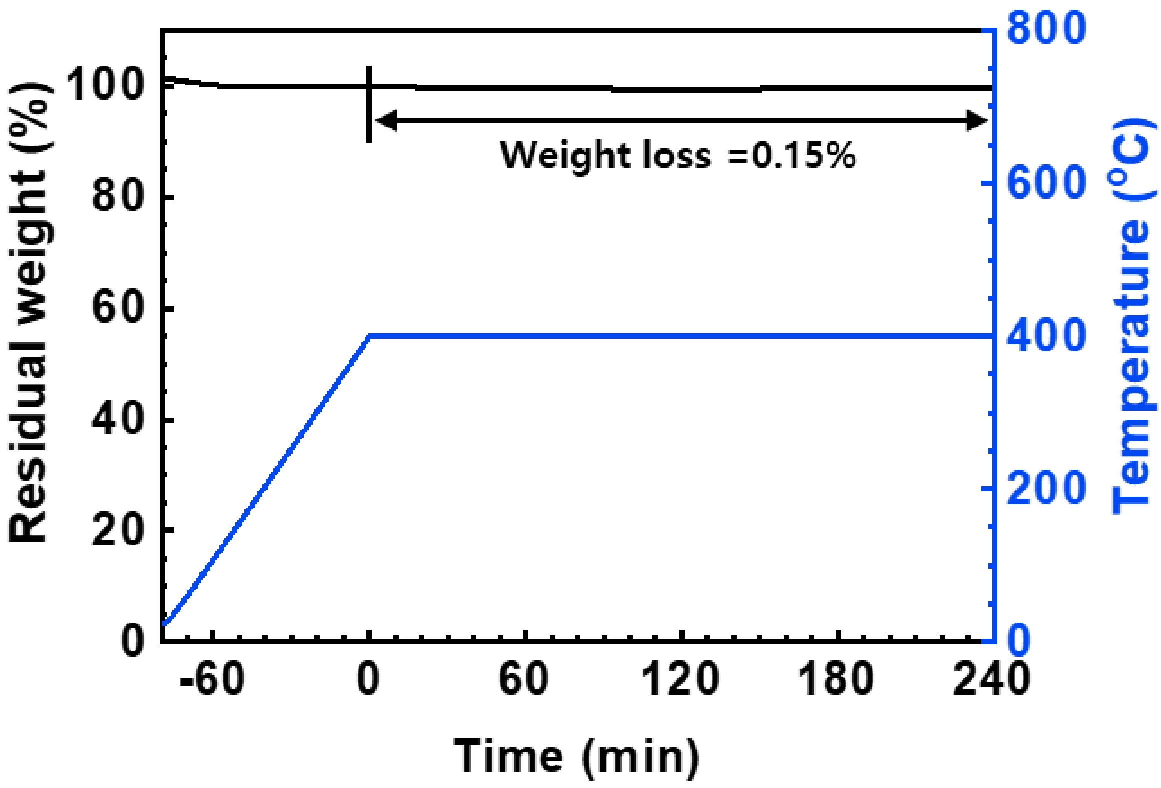

Supplementary Fig. 9. Isothermal TGA curve of TR-PBO at 400 °C

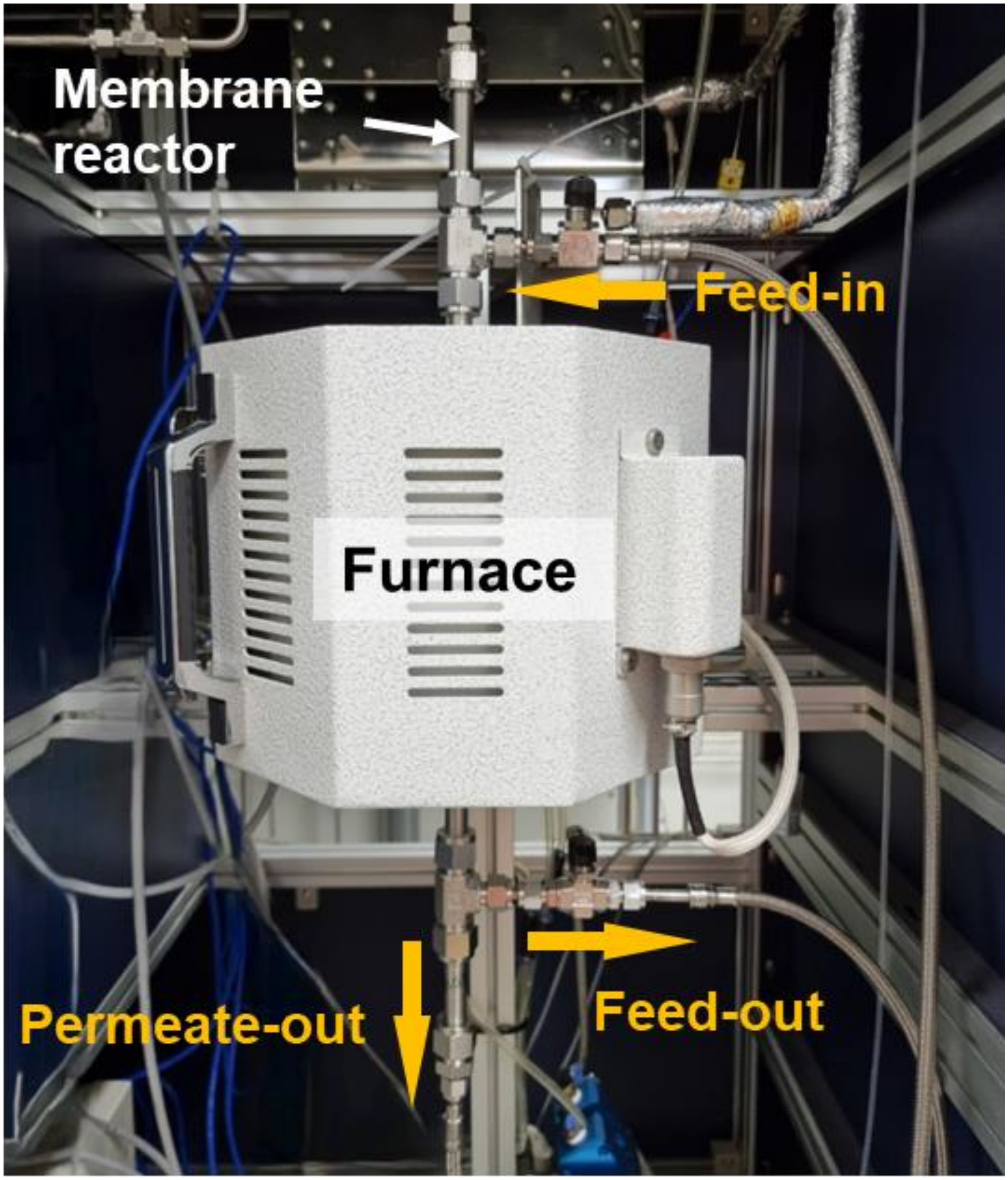

Supplementary Fig. 10. Setup for the gas permeation test

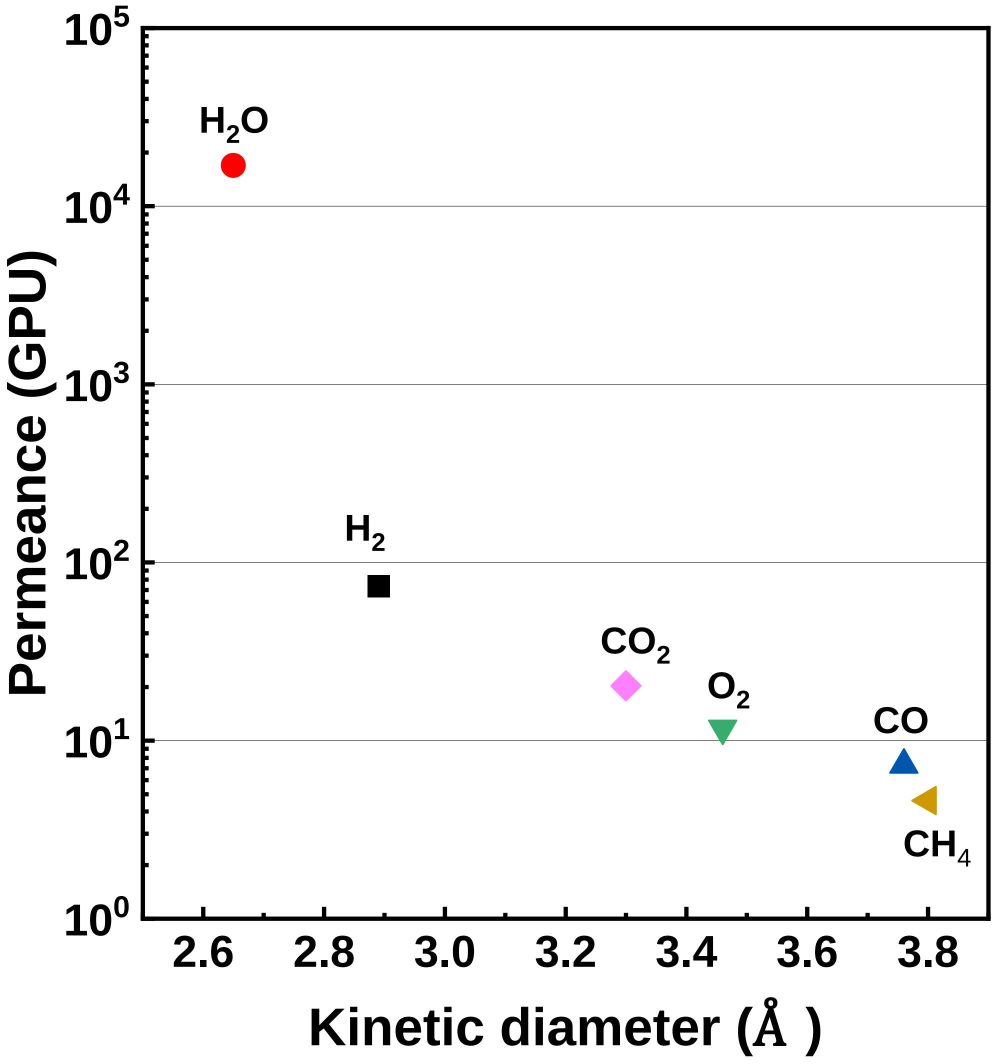

Supplementary Fig. 11. Comparison of permeance of gases at 350 °C according to the kinetic diameter of molecules

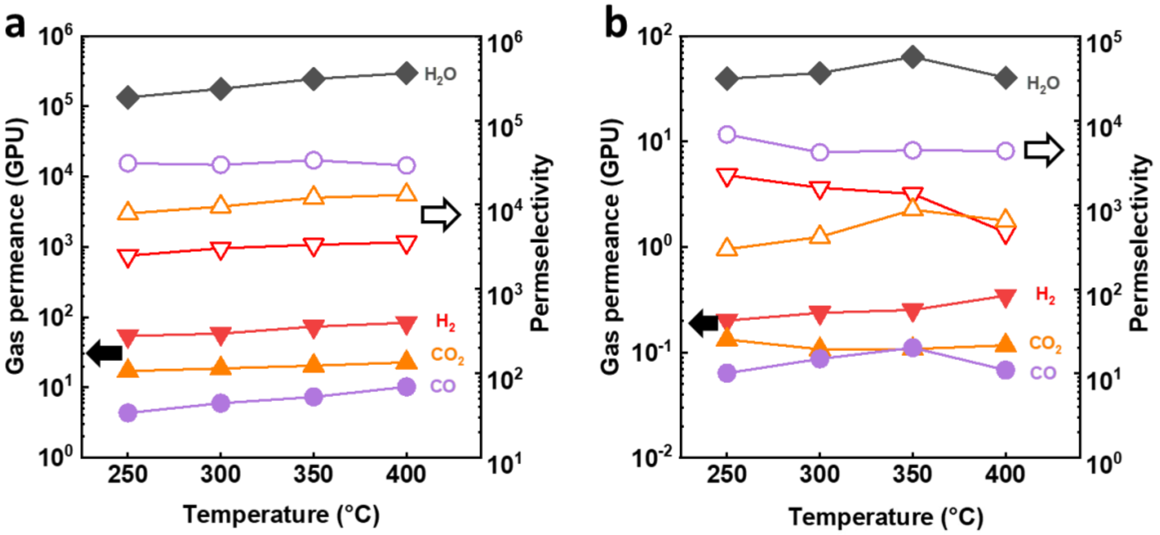

Supplementary Fig. 12. Comparison of permeabilities of **a** single gas and **b** mixed gas. The solid and hollow symbols denote the permeance and H_2O/gas permselectivity, respectively. The mixed gas permeation test was performed using RWGS reaction setup, where sweep gas was not fed.

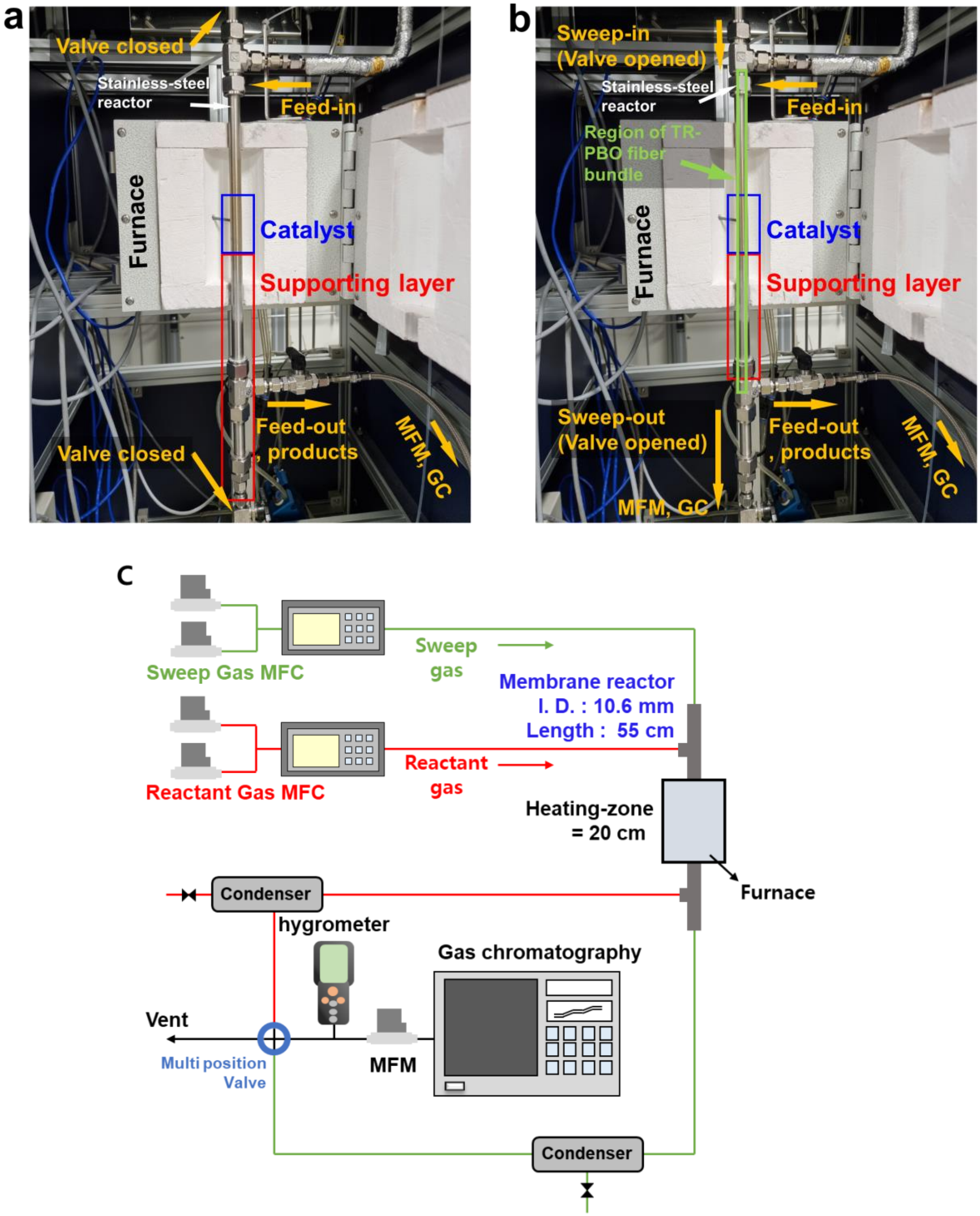

Supplementary Fig. 13. Setup for testing catalytic reaction: **a** neat catalyst and **b** membrane-catalyst hybrid system. **c** Schematic of the overall reactor

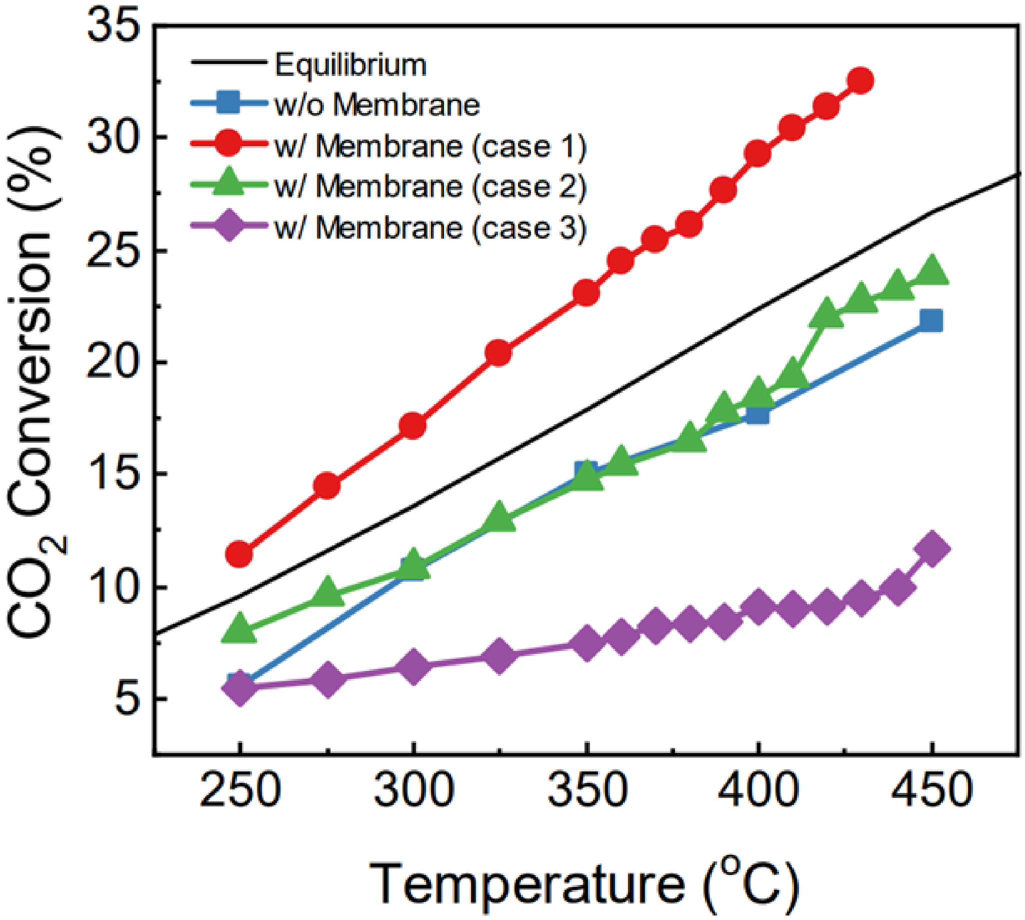

Supplementary Fig. 14. CO_2 conversion in the RWGS reaction conducted using the TR-PBO membrane reactor with different sweeping gases. Flow rates of reactant gas (H_2/CO_2=1) fed into the catalyst part were fixed at 24 sccm for all cases: Case 1: flow rate of the sweeping gas (H_2/CO_2=1) was 24 sccm. Case 2: Sweeping gas was not fed. Case 3: Flow rate of the sweeping gas (N_2) was 6 sccm

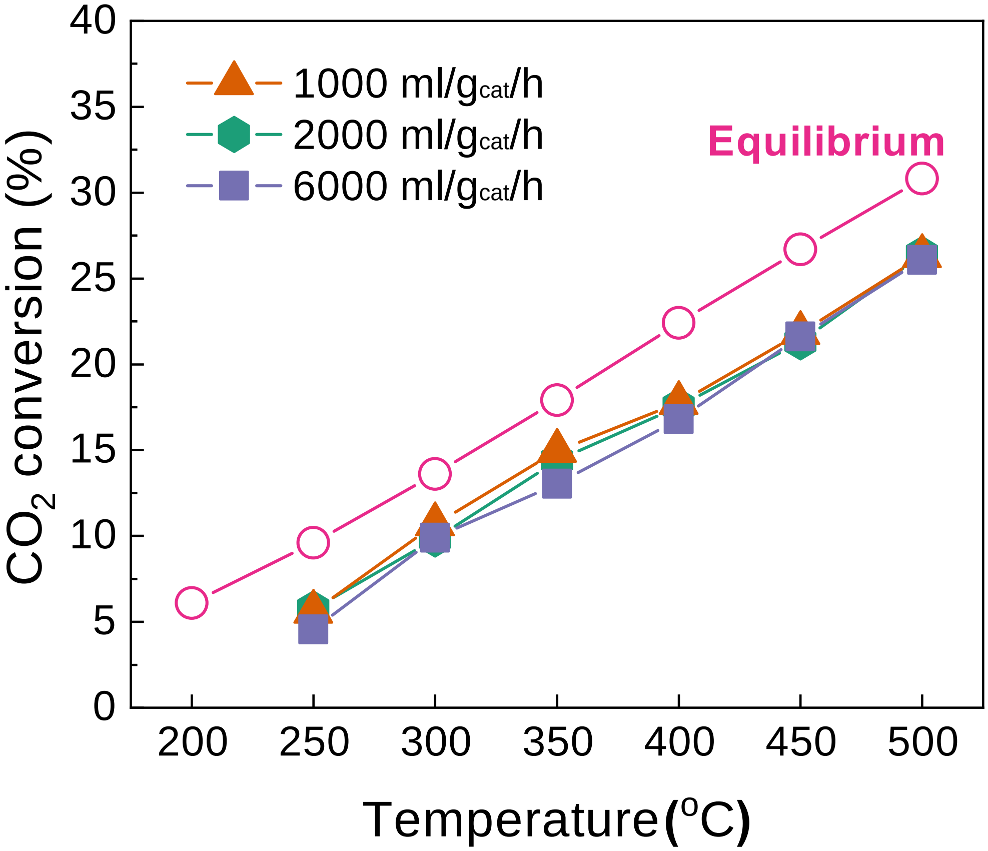

Supplementary Fig. 15. CO_2 conversion in the RWGS reaction conducted using a fixed-bed reactor without the membrane. The reactions were tested at three different space velocities: 1000, 2000, and 6000 mL/g_{cat}·h. The reactor has dimensions identical to that of the membrane reactor, including the catalyst bed configuration. The CO_2 conversion was almost the same under all three conditions, suggesting that all the reactions occurred close to the experimental equilibrium, which is 3–5% lower than the theoretical equilibrium conversion (blank circles). Because a lower space velocity was applied in the membrane reactor, the increase in the CO_2 conversion in the membrane reactor is attributed to the equilibrium shift due to H_2O removal.

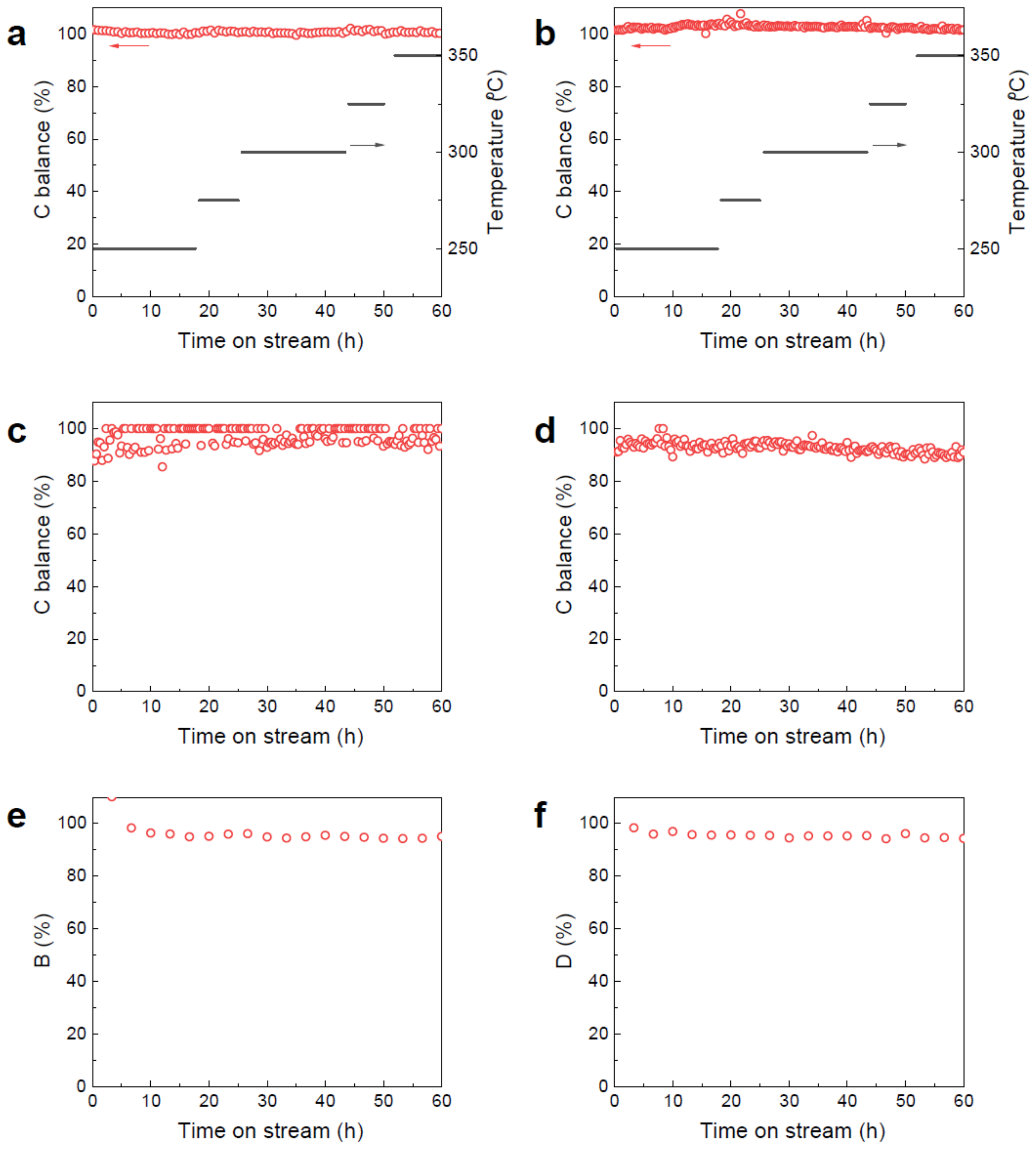

Supplementary Fig. 16. Carbon balances of the **a** RWGS with the membrane, **b** RWGS without the membrane, **c** CH_4 oxidation with the membrane, **d** CH_4 oxidation without the membrane, **e** FTS with the membrane, and **f** FTS without the membrane. The carbon balance calculations for the RWGS, CH_4 oxidation, and FTS reactions are provided in equations (7), (11), and (17), respectively.

Supplementary Tables

Supplementary Table 1. Spinning conditions for fabricating hollow fiber

Dope composition (HPI/NMP/THF wt. ratio)	23/62/15
Dope flow rate (mL/min)	1.0
Bore flow rate (mL/min)	1.0
Spinneret temperature (°C)	55
Coagulation bath temperature (°C)	25
Air gap (cm)	20
Coagulant	Tap water
Bore fluid	DI water

Supplementary Table 2. Comparison of the volume of TR-PBO integrated and packed-bed reactors for 1000 kmol/h production of CO. RWGS reaction temperature of 400 °C and the effective membrane area of 2.28×10^5 m^2 are assumed based on the lab-scale reaction result. Reactor tube dimension: height = 11 m; diameter 4 inch. The water permeation rate at the reaction temperature of 400 °C allows the maximum feed flow of 3024 mL/g_{cat}/h for the TR-PBO membrane reactor, while feed flow of 3000 and 6000 mL/g_{cat}/h are applied to the conventional packed-bed reactor.

TR-PBO membrane reactor specification				
Water permeation rate @ 400 °C			mL/min/cm^2	0.56
Water removal ratio			%	46
Maximum feed flow			mL/g_{cat}/h	3024
Effective membrane volume per catalyst weight			cm^3/g	0.0689
Specific volume of catalyst			cm^3/g	0.79
Comparison				
		Membrane reactor	Packed-bed reactor	
CO production rate	kmol/h	1000	1000	1000
GHSV	mL/g_{cat}/h	3000	3000	6000
CO_2 conversion	%	27	15	15
Catalyst weight	kg	55308	99555	49777
Catalyst loading per tube	kg	100.5	109.3	109.3
Number of reactor tubes		551	911	455

.

Supplementary References

[1] Kang, S. C., Jun, K.-W. & Lee, Y.-J. Effects of the CO/CO2 ratio in synthesis gas on the catalytic behavior in Fischer–Tropsch synthesis using K/Fe–Cu–Al catalysts. Energy & fuels 27, 6377-6387 (2013).

[2] Lee, J. et al. Low-temperature CO2 hydrogenation overcoming equilibrium limitations with polyimide hollow fiber membrane reactor. Chemical Engineering Journal 403, 126457 (2021).

[3] Jo, H. J. et al. Thermally rearranged poly (benzoxazole-co-imide) membranes with superior mechanical strength for gas separation obtained by tuning chain rigidity. Macromolecules 48, 2194-2202 (2015).

[4] Pope, C. G. X-ray diffraction and the Bragg equation. J. Chem. Educ. 74, 129 (1997).

[5] Woo, K. T. et al. Ternary mixed-gas separation for flue gas CO2 capture using high performance thermally rearranged (TR) hollow fiber membranes. J. Membr. Sci. 510, 472-480 (2016).

[6] Li, H. et al. Na+-gated water-conducting nanochannels for boosting CO2 conversion to liquid fuels. Science 367, 667-671 (2020).

[7] Rezai, S. A. S. et al. Water/hydrogen/hexane multicomponent selectivity of thin MFI membranes with different Si/Al ratios. Microporous Mesoporous Mater. 108, 136-142 (2008).

[8] Sawamura, K. i. et al. Reverse-selective microporous membrane for gas separation. Chem. Asian J. 4, 1070-1077 (2009).

[9] Espinoza, R. et al. Use of membranes in Fischer-Tropsch reactors. Stud. Surf. Sci. Catal. 130, 389-394 (2000).

[10] Raso, R. et al. Zeolite membranes: Comparison in the separation of H2O/H2/CO2 mixtures and test of a reactor for CO2 hydrogenation to methanol. Catal. Today. 364, 270-275 (2021).

[11] Sawamura, K.-I. et al. Selective permeation and separation of steam from water–methanol–hydrogen gas mixtures through mordenite membrane. Catal. Today. 132, 182-187 (2008).

[12] Zhu, W. et al. Water vapour separation from permanent gases by a zeolite-4A membrane. J. Membr. Sci. 253, 57-66 (2005).

[13] Lee, S. M. et al. Structure, stability and permeation properties of NaA zeolite membranes for H2O/H2 and CH3OH/H2 separations. J. Eur. Ceram. Soc. 38, 211-219 (2018).

[14] Aoki, K., Kusakabe, K. & Morooka, S. Separation of gases with an A-type zeolite membrane. Ind. Eng. Chem. Res. 39, 2245-2251 (2000).

[15] Gorbe, J. et al. Preliminary study on the feasibility of using a zeolite A membrane in a membrane reactor for methanol production. Sep. Purif. Technol. 200, 164-168 (2018).

[16] Sjöberg, E., Sandström, L. & Hedlund, J. Membrane processes for effective methanol synthesis in the forest based biorefinery. Catal. Today. 156, 87-92 (2010).

[17] Sato, K. et al. Application of FAU-type zeolite membranes to vapor/gas separation under high pressure and high temperature up to 5 MPa and 180 C. Microporous Mesoporous Mater. 101, 312-318 (2007).

[18] Wang, N., Liu, Y., Huang, A. & Caro, J. Hydrophilic SOD and LTA membranes for membrane-supported methanol, dimethylether and dimethylcarbonate synthesis. Microporous Mesoporous Mater. 207, 33-38 (2015).

[19] Lafleur, M. et al. Development of a water-selective zeolite composite membrane by a new pore-plugging technique. Microporous Mesoporous Mater. 237, 49-59 (2017).
